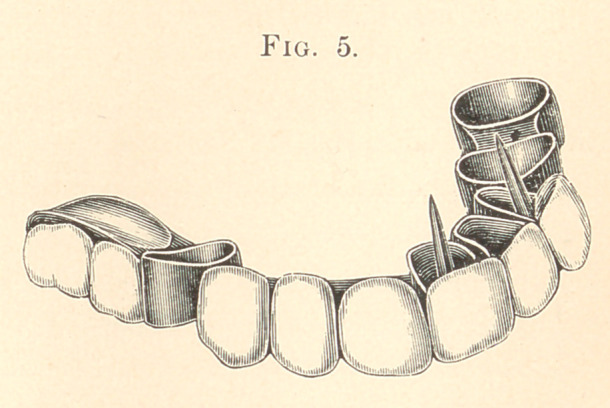# Crown- and Bridge-Work

**Published:** 1892-08

**Authors:** C. M. Richmond

**Affiliations:** New York


### CROWN- AND BRIDGE-WORK.[1]

BY DR. C. M. RICHMOND, NEW YORK.

(Continued from page 489.)

In this, my second article on crown- and bridge-work, I have shown an upper set of teeth with the attachments all on one side of the mouth, except one bicuspid, which makes it a very interesting operation, unlike a piece of bridge-work where the points of attachment are evenly distributed, or nearly so. We must study the case and see how we can make the work so it will be held firmly in position, and not draw on some of the teeth more than others, and so ruin the whole structure. In this operation, like my first one, we must primarily have perfect-fitting attachments, bands, and posts, and the whole must, when in position, fit so exactly as to be immovable in any part of the structure.

This operation has a double interest, as it has been through all of the evolutions from single crowns, with a plate to fill out the set; then for ten years wearing a fixed bridge. But now comes a beautiful movable bridge, to take the place of all the old experiments; and the operation is, after all the trials of the preceding cases, a monument to the principle upon which it is constructed.

The two bicuspids and molars have gold crowns, and are the first ones put in the mouth by Dr. E. O. Cochrene, 850 Market Street, San Francisco, eighteen years ago; and the method he then employed was making a gold band to fit a metallic die of each

---

[1] Copyright, 1892, by Dr. C. M. Richmond.

tooth, then soldering a flat top on to each band, and making each cusp of solid gold and soldering into position, one at a time. This was slow and tedious work, compared to the method now used for making gold crowns, but the crown is beautiful and durable, and has thickness enough so that it can be ground and articulated perfectly, without the danger of cutting through. This method of construction has made it possible to use the same old crowns through all the changes that have been made in this case for the past eighteen years.

Referring to the cuts, Fig. 1 shows the cap and square tube with top or piece in position, ready to receive the porcelain tooth as shown in Fig. 2. Each of the roots, as shown in the cut, Fig. 3, have been fitted with the band, which is bevelled on the facial surface, the same as if it were to be made into the original Richmond crown method.

The top is covered with two pieces of plate; that is, I solder first the flat part on to the band, then the bevelled surface piece is soldered into place. I have now a gold cap that fits the roots perfectly, and having previously made some square tapering gold tubes (which in turn receive a perfectly-fitting iridio-platinum pin), I wax them into the square holes in the top of the gold cap, trying each one into its respective root to see if they are in the right position to allow the bands to go down to their places after they are perfectly adjusted. I then take each one and invest it in the ordinary way, fitting the square tube with a little of the investment far enough to prevent the solder from running into or making the tube imperfect. The least solder will ruin the perfect fitting of the pin into the tube.

The greatest care must be used in each step, or the work will not be what we term "working to the line;" no half-way guesswork will do; everything must be perfect.

After each cap is fitted on to the roots, with the tube in as shown in Fig. 3, I now grind the teeth into position on each bevelled surface on the caps, while they are on the roots; the length of tooth, the pitch, in or out, the irregular position, if it is desired, are all determined here. The caps are now removed and the pin and half-caps are made to slip into position waxed together, as shown in Fig. 1.

The half-band is now bent to fit the inner surface of the cap, and the cap is soldered on to it, being careful not to use solder enough to allow of any imperfection by the solder running inside of the half-band. The pin and half-band must be invested and

FIG. 1.

FIG. 2.

FIG. 3.

FIG. 4.

FIG. 5.

solder enough to catch the pin to the half-band, and then we are ready for the porcelain.

I have referred to grinding the teeth to the caps in the mouth; the case can now be waxed so that it looks like Fig. 2, and has the same appearance after it has been invested, and the tooth soldered to the pin and and half-cap.

After these three teeth are finished, as in Fig. 2, I put each tooth into position on my caps and cement them in said position, and after I have removed the three teeth we have the case as shown in Fig. 3.

This case is now ready for the teeth. The molars and bicuspids and gold crowns are a trifle smaller at the cutting edge, so that when an impression of them is taken and our metallic dies, as shown in Fig. 4, are made, we can make a telescopic band that will go on to the place when it is fitted and will go no farther; so that when we have the work done and put into position, each band and each pin and half-band will all stop and have their perfect bearing at the same time.

Between the teeth (the incisor and bicuspid) is a bridge, but beyond the bicuspids I put the case on to a perfectly-fitting small gold plate.

The mode of procedure from this point is to take an impression of the case with the three teeth in position; the teeth will come out with the impression, and, after fitting the impression with the investment, the plaster is cut away and the teeth are perfectly covered with more investment, and these three teeth are soldered together. These can now be put on and taken off while the three teeth are being waxed into position to fill the space from the centre to the bicuspid after the six front teeth are all right and in their proper position.

I now place the two gold bands previously made for the bicuspids into place, with the six front teeth also in position. I now take a plaster impression over the case, and after filling the impression (with the two gold bands and the six front teeth in it) with investment, cut away the plaster and cover the teeth with more investment, and solder the case. We have now the six front teeth and the two gold bands in one piece. The object of putting this case together in detail is to insure a perfectly-fitting piece of work. Where so much soldering and so many teeth are put together in one piece it is not possible to arrange and solder it as a whole and be successful.

After the work is soldered to this point it is put into position, and the gold band is placed on the molar and an impression is taken of the case (being careful to get it beyond the bicuspids), where we are to fit a small plate to the gum, on which we are to place two more teeth, and solder the whole together in one piece, as shown in Fig. 5.

In making the plate to carry the two teeth, I use a piece of pure gold (30 thick, U. S. gauge), and it can be burnished to fit the cast with slight pressure. I then back up two plain molars with pure gold and wax them into position, and the soldering is done in the usual way.

The last gold band is soldered to the case at the same time the two molars are finished. The case will now go on to the gold crowns, and a perfect fit is the result if all the details are carefully carried out. This case, after years of wear, is as firm and perfect as if it were made yesterday. All the pathological conditions must be understood in these extensive operations, as it is *imperative* to complete success.

I will now give my method of management of all classes of teeth and roots that we are called upon to treat pathologically.

When first beginning the practice of crown-work as a specialty, my mode of treatment of these roots was similar to that followed by the profession for twenty years. But from rapidly-accumulating operations, the necessary delay in the treatment of teeth termed dead, or those with devitalized nerves, was very annoying, not only to myself but to my patients.

The mode which I here propose to give was brought about entirely by chance. I had occasion to excise a tooth which had a live nerve in it at the time, upon the root of which I wished to place a piece of bridge-work, this tooth to be one of the abutments. The operation was one that required three or four sittings; and after the crown had been excised, and the live pulp extirpated, in the manner which I shall speak of hereafter, the canal of this root was left open. The next morning the patient came to the office, suffering from very severe acute periosteal inflammation; exactly the condition of things that we expect the next day after cutting into a dead tooth, so called, or removing the pulp from such a tooth.

This demonstrated to me the fact that this condition of things was not due in either case to the previous death of the pulp, but was brought about by the presence of the atmosphere in the pulp-canal and its penetration through the foramen into the soft tissues.

From this discovery, I commenced my experiments in the management of crownless roots, and I treated them in exactly the same manner, no matter what their previous condition had been, the result being universally satisfactory.

I classify the different conditions that I find existing in teeth, where the pulps have been previously devitalized, by first giving a description of my mode of treatment of a tooth where it is necessary to remove the pulp when in a healthy condition.

In practising the crown- and bridge-work, as hereinbefore set forth, it has often been necessary to sacrifice a good crown for the sake of utilizing the root in the operation ; and so well satisfied am I of the result of these operations that I never hesitate for a moment to excise the living tooth for the purpose of this attachment.

In excising the living tooth I always make an application of nerve-paste. Twelve hours later a crown so treated can be removed and the pulp extirpated without pain. After removing the crown and extirpating the pulp, I immediately wash out the canal with carbolic acid, and provide a piece of orange-wood whittled down to fit the shape of the pulp-canal. After being thoroughly saturated with carbolic acid this wooden plug is driven to its place in the canal by a slight tap of a mallet. A tooth treated in this manner will remain in the mouth as long, and do as good service, as its nearest neighbor with a living pulp.

I arrange these teeth as follows, and give the treatment persued in each class.

*First*, teeth the pulps of which have become devitalized, from whatever cause, and which had never given the patient trouble, or indications of it; the pulps still living.

*Second*, teeth where the pulp is dead, and a dormant abscess exists at the apex of the root, but from which the patient has had nothing more than a slight feeling of something abnormal existing there.

*Third*, roots where the pulp is dead, and an abscess has formed with a fistula externally through the process and gum-tissues.

*Treatment* of first class. Whether these cases are treated by removing the crown or through the cavity which may have been formed by decay, or been opened with the drill, I first apply, after the pulp-chamber has been thoroughly exposed, a drop of carbolic acid, through which must pass whatever instrument I use for cleaning the pulp-canal. I then very carefully follow up into the canal with the instrument (usually a delicate piece of orange-wood whittled to shape for the purpose); the carbolic acid all the time closing the mouth of the canal completely, and thereby preventing the atmosphere from entering it. In this way it is soon thoroughly cleansed without pressure, and with very delicate manipulation; every particle of the decayed pulp being removed. The piece of orange-wood is now driven into the canal and cut off, in precisely the same manner that I have described in the extirpation of the live pulp.

Treatment of the second class, or roots where the pulp is dead and a dormant abscess exists without a fistula. This class of cases is generally found in the mouths of people of robust constitution, and the circulation carries off the blood and no fistula forms. These teeth should be treated in precisely the same manner as the preceding class, great care being exercised during the cleansing of the pulp-canal to thoroughly exclude the atmosphere by the application of carbolic acid.

Treatment of the third class, or teeth where abscesses have formed as the result of dead pulp in the roots, and there being a fistula through the gum. These cases are treated precisely as those already described, except that a rose-burr is introduced through the fistula to the end of the root, and a dressing of cotton lint saturated with carbolic acid left there as a tent. These cases will almost always recover with this treatment; whereas, if the dentist, in his anxiety to cure the lesion by the ordinary treatment, pumps into the fistula iodine, aconite, carbolic acid, creosote, iodoform, etc., and then carries the case to the local society with its history and asks, What else shall he do to effect a cure? The answer comes from one having had similar experience, "Give it a rest!" The first faint shadow of good sense then creeps into the brain of the operator, and he sees that, in his anxiety to cure the case, he has put it into a worse condition than it was at first by *over*-treatment.

I will now give the history of some noted cases, which were operated upon by myself in New York for the purpose of demonstrating to dentists, among whom were Dr. J. L. Williams and others, who had witnessed the uniform success of my treatment and had seen the failure of the old methods. The first case I shall describe is that of a gentleman who, from a blow, had the six inferior front teeth partially driven out of their sockets, so that the pulps were severed, and when these teeth were pressed back to their position and had become firm again, they were very much discolored, and the gentleman wished to have them replaced with artificial crowns. He was of such a robust constitution that the teeth had never given him any trouble, even so much as an acute inflamation would produce, to say nothing about a dormant abscess or a fistula, so that it was a satisfactory case for the demonstration of my theory; and I said to the gentlemen in my office that I

would here prove to them the uniform success of my management of such teeth, and at the same time demonstrate how useless the methods ordinarily used. I took five of the six teeth and thoroughly cleansed the pulp-canals as I have described, being careful that no atmosphere should penetrate into them, and they were all filled at one sitting with a piece of orange-wood whittled to the shape of the pulp-canal, as heretofore described, and driven in with a mallet; the sixth tooth I cut open and treated in the ordinary way, using no carbolic acid to prevent the atmosphere from penetrating into the canal, but cleansing it thoroughly and packing it lightly with cotton and carbolic acid; treating it exectly as such teeth are usually treated by the dental profession. The result was that the next day the five teeth which I had handled according to my method, or in the manner described above, gave no signs of soreness or trouble, while the one which had the cotton lint and the carbolic acid, and was left open, developed an abscess; the man's face was very much swollen, requiring an opening made with a drill to relieve it. The results of these different modes of treatment of teeth in the same condition and in the same mouth and at the same time demonstrate the superiority of my mode of treatment, and that there is no mistake about the uniform good results claimed for it.

Another case which I will describe is that of a person of robust constitution, who desired to have some artificial crowns placed upon a number of roots in the upper jaw, which had been excised and filed down even with the gum for the purpose of putting in a gold plate.

I found the nerve-canal of these ten superior teeth filled with debris of dead pulp and other foreign matter. I treated them the very first time I met the patient in the manner above described. The next day, when the gentleman called, he was suffering no inconvenience whatever, and the operation of placing crowns upon these roots was at once commenced. As fast as a crown was set, a tooth was cut from the gold plate to allow it to go back to its original position, and the operation was continued. The third day after the operation was commenced and the pulp-canals were filled, a slight soreness was complained of in the canine tooth, which had been crowned the day previous. I inquired of the patient whether he had ever had any trouble from it before, and found that he had sometimes felt a stiffness, as he termed, in that immediate locality. The gum and process were at once punctured to the apex of the root, and the next day he was perfectly happy. I placed in this mouth twenty-six artificial crowns. The roots were all treated in precisely the manner which I have above described. The case is now of thirteen years' standing, and I have never had the least complaint from this patient in regard to any of those teeth.

In conclusion, concerning the theory of my treatment, I will say that it is only carrying out the same ideas that are now followed in latter-day surgery, in this respect, that all forms of animal life in the atmosphere are entirely excluded by the use of a spray of carbolic acid, listerine, or other well-known applications, thereby causing the wounds to heal without suppuration. I therefore simply advocate the prevention of the cause of the trouble. Let the manipulations be carefully and delicately performed, and the result will be most satisfactory, and all who try the mode which I have here described will have no cause to regret changing their method in the treatment of such teeth. I can also add that all those that have been doing this for years are satisfied with it, and those who will adopt it can be made happy, and their patients thereby saved the annoyance of a most disagreeable experience by lack of knowledge of a better mode.

## Figures and Tables

**Fig. 1. f1:**
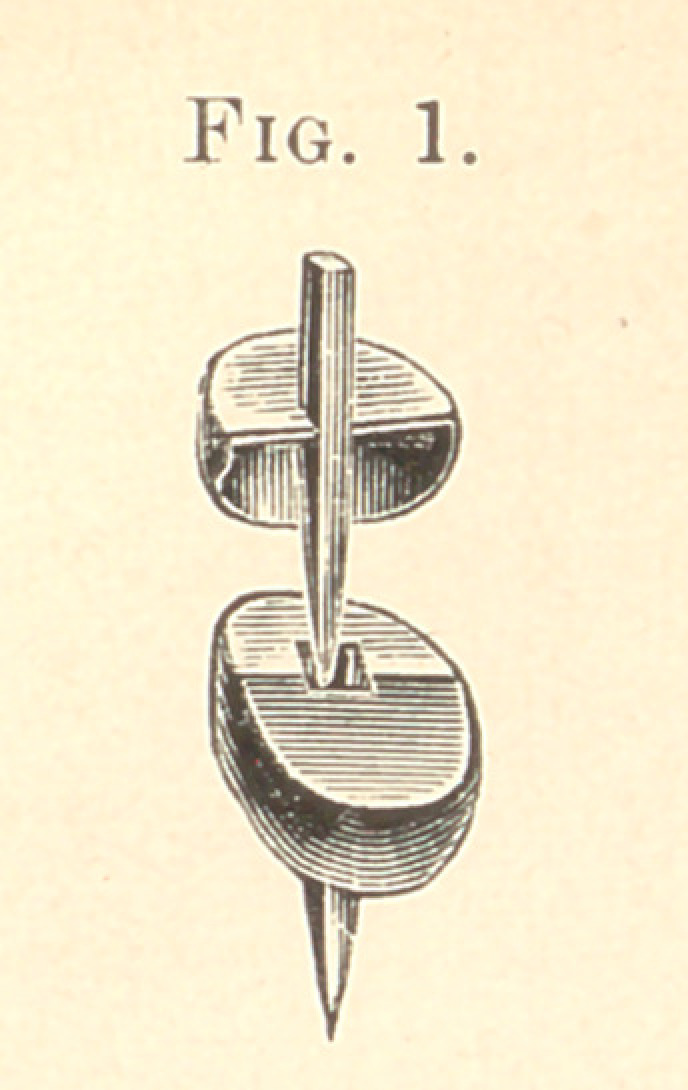


**Fig. 2. f2:**
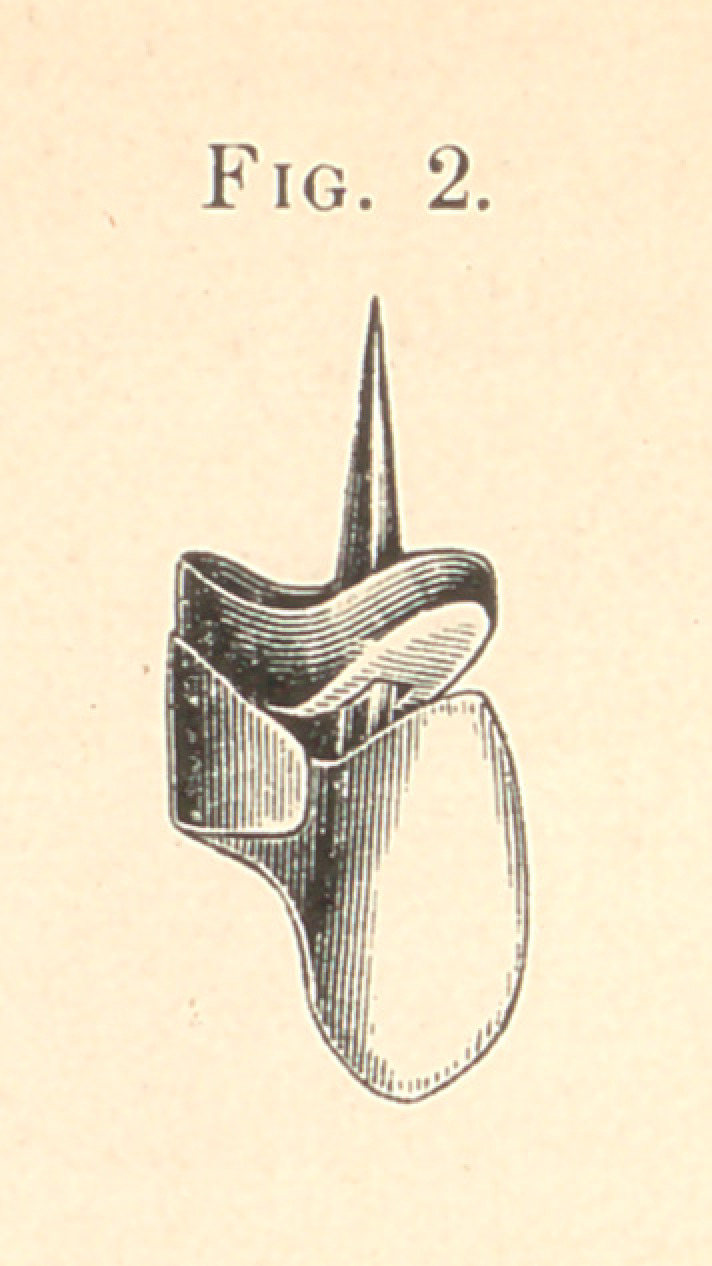


**Fig. 3. f3:**
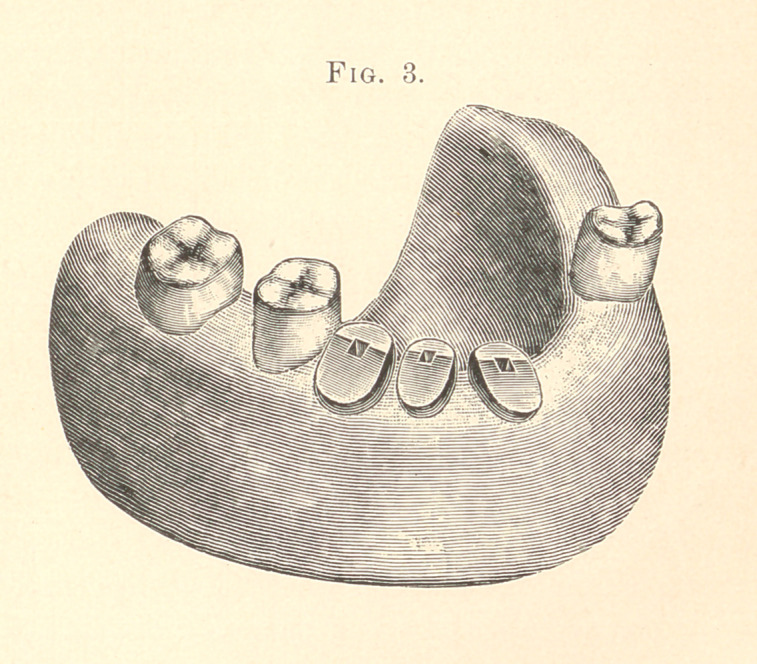


**Fig. 4. f4:**
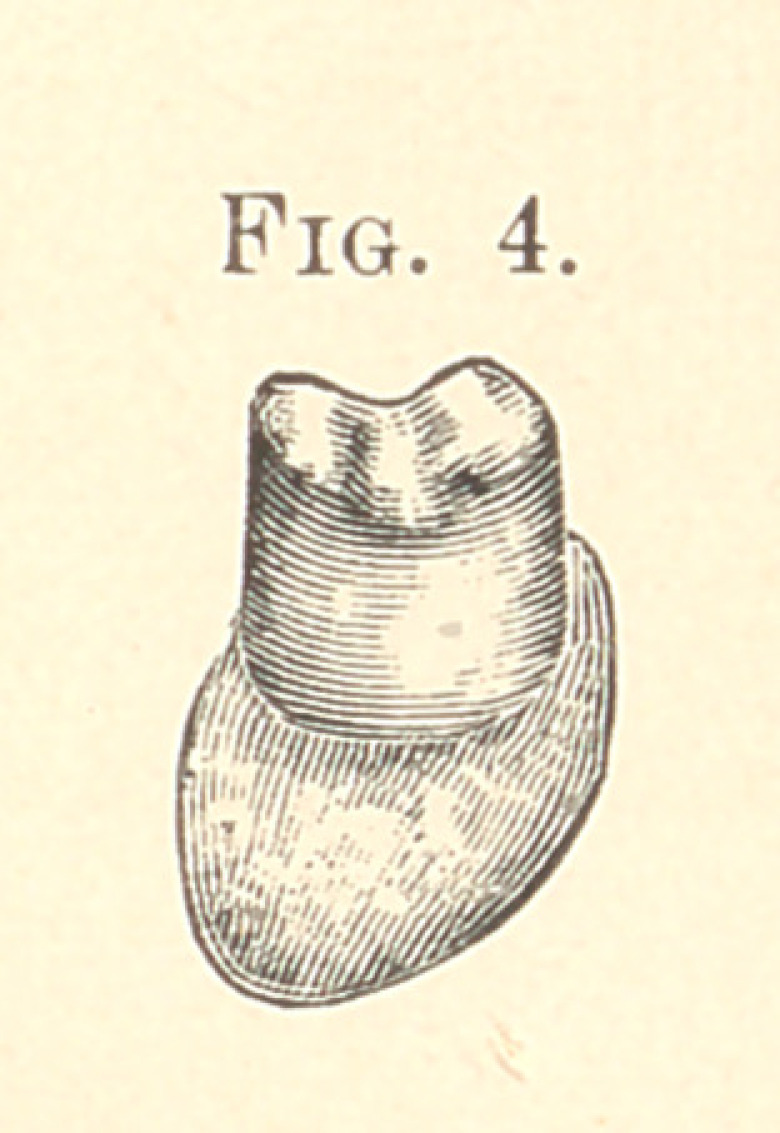


**Fig. 5. f5:**